# Marital conflict resolution through schema therapy and mutual behaviour analysis for enhanced self-differentiation in Kwara State

**DOI:** 10.3389/fsoc.2026.1866983

**Published:** 2026-07-14

**Authors:** Lateef Omotosho Adegboyega, Adeola Aminat Odebode, Samuel Kolawole Ajiboye, Alfred Olatayo Awoyemi, Segun Olayide Odutayo, Abdulrasaq Olatunji Balogun

**Affiliations:** 1 Department of Educational Guidance and Counselling, Faculty of Education, University of Ilorin, Ilorin, Nigeria; 2 Department of Social Sciences Education, Faculty of Education, University of Ilorin, Ilorin, Nigeria

**Keywords:** Kwara state, marital conflict resolution, mutual behaviour analysis, schema therapy, self-differentiation

## Abstract

**Introduction:**

Marital conflict remains a persistent challenge among couples in Nigeria, often driven by deep-rooted maladaptive cognitive schemas, dysfunctional behavioural patterns, and poor self-differentiation. This study examined marital conflict resolution through Schema Therapy and Mutual Behaviour Analysis for enhanced self-differentiation among married couples in Ilorin Metropolis, Kwara State.

**Methods:**

A quasi-experimental pretest–posttest control group design was adopted, involving 45 married couples. The instruments used were the Maladaptive Schema and Behavioural Patterns in Marital Conflict Scale (MSBPMC) and the Self-Differentiation Questionnaire (SDQ). Data were analyzed using mean, standard deviation, and ANCOVA at 0.05 level of significance.

**Results:**

Findings revealed that emotional deprivation, abandonment/insecurity, and unrelenting standards were the most dominant maladaptive schemas, while demand–withdraw, emotional withdrawal, and avoidance behaviours were most prevalent. Couples also exhibited low self-differentiation characterized by emotional reactivity and dependence. Both Schema Therapy and Mutual Behaviour Analysis significantly reduced marital conflict and improved self-differentiation, with Schema Therapy showing a slightly greater effect. The combined ST+MBA model was most effective.

**Discussion and recommendations:**

The study concludes that integrative therapeutic approaches are essential for effective marital conflict resolution.

## Introduction

Marriage is a fundamental social institution that binds individuals in a legally and culturally recognized union, primarily for companionship, procreation, and mutual support. It serves as the bedrock of family life and plays a crucial role in maintaining social order and continuity within society. According to Olatunji (2022), marriage is a culturally sanctioned relationship that establishes enduring bonds between partners and provides a framework for family formation and social continuity. Similarly, Yusuf and Adewale (2023) described marriage as a union characterized by emotional attachment, shared responsibilities, and societal approval, which legitimizes cohabitation and procreation. In many societies, including Nigeria, marriage extends beyond the couple to include their families and communities, reflecting deeply rooted traditions, values, and belief systems that shape spousal roles and expectations. Consequently, marriage is viewed as a partnership that requires cooperation, commitment, and mutual understanding for stability and harmony. Its success largely depends on how well couples adjust to individual differences and fulfill relational roles (Adebayo and Oladipo, 2023).

Despite its significance, marriage is not without challenges, and marital conflict remains a common feature of intimate relationships. Marital conflict refers to disagreements, tensions, or disputes that arise between spouses due to differences in opinions, needs, values, or expectations. Ogunleye and Afolabi (2022) defined marital conflict as a process in which partners experience opposition or incompatibility in their interactions, often resulting in emotional strain. Such conflicts may arise from financial difficulties, ineffective communication, child-rearing practices, and unmet expectations within marriage. These disagreements are often inevitable, given that partners come from different backgrounds and may hold differing perspectives on important aspects of life (Yusuf and Adewale, 2023).

In Nigeria, marital conflict has become increasingly recognised across socio-economic and cultural groups. Couples frequently encounter disagreements arising from financial pressure, communication difficulties, sexual dissatisfaction, and interference from extended family systems. James and Egwuatu (2024) affirmed that such conflicts are not rare occurrences but form part of everyday marital experiences. Similarly, Ogunwale and Afolabi (2022) reported notable levels of coercive and conflict-related behaviours among married women in South-West Nigeria, indicating that marital tension remains a significant challenge affecting family stability and well-being.

In Kwara State, marital conflict is also evident, although it is often underreported due to cultural expectations that discourage open discussion of family matters. Studies focusing on married adults in the state indicate that economic hardship, unemployment, and inability to meet family responsibilities are major contributors to conflict between spouses (Logun et al., 2023). These stressors often generate tension that manifests as frequent disagreements and emotional distance within marriages. Similarly, Ogungbemi et al. (2024) reported that newly married couples in Ilorin commonly experience conflict due to poor communication skills, unmet expectations, and external interference from relatives, suggesting that marital conflict often emerges early in many unions.

Although marital conflict is prevalent, not all disagreements escalate into severe forms such as domestic violence. Evidence from Kwara State suggests that while conflict is widespread, its intensity varies. Ajayi and Bello (2024) observed that different forms of disagreement such as verbal and financial disputes are common among married adults, but extreme violence is not uniformly prevalent. This implies that many couples experience moderate levels of conflict that may still affect marital satisfaction and stability if not properly managed. Further evidence links relational dissatisfaction to ongoing marital disagreements. Issues such as sexual dissatisfaction and extramarital involvement have been identified as significant predictors of marital tension among couples in Kwara State (Adebayo et al., 2025; James and Egwuatu, 2024). These factors often weaken emotional bonds and increase the likelihood of persistent conflict between partners.

The manner in which couples handle marital conflict plays a crucial role in determining the quality and longevity of their relationship. Constructive conflict resolution strategies such as open communication, empathy, negotiation, and mutual respect can promote understanding and strengthen marital bonds. In contrast, destructive strategies including hostility, avoidance, and persistent criticism are associated with dissatisfaction and instability. Empirical evidence suggests that couples who adopt positive conflict management strategies tend to experience higher levels of marital satisfaction and overall well-being (Falade and Eze, 2022; Williamson et al., 2023). This highlights the importance of therapeutic approaches that enhance partners’ awareness of interaction patterns and promote healthier ways of managing disagreements.

Within this context, Schema Therapy provides a useful framework for understanding how early life experiences shape individuals’ interpretations and responses to marital conflict. It is based on the premise that individuals develop deep-seated cognitive and emotional patterns known as schemas during childhood, particularly when core emotional needs are unmet. These schemas may be activated in adult intimate relationships, leading partners to respond to present-day marital issues through the lens of past emotional injuries. For example, feelings of abandonment or mistrust may trigger exaggerated emotional reactions during minor disagreements. Schema Therapy therefore helps couples identify maladaptive schemas, challenge distorted beliefs, and develop healthier emotional and cognitive responses that improve relational functioning (Young et al., 2003).

Similarly, Mutual Behaviour Analysis focuses on how the actions of one partner influence and reinforce the behaviour of the other within the relationship. It is grounded in the understanding that marital interactions are reciprocal and cyclical rather than one-sided. Negative behaviours such as criticism, defensiveness, or withdrawal often form part of an interactional cycle that escalates conflict. By examining these patterns, couples gain insight into how their responses contribute to relationship distress. This awareness provides opportunities to interrupt negative cycles and replace them with constructive behaviours such as active listening, validation, and cooperative problem-solving, thereby improving communication and reducing emotional tension (Dattilio, 2010).

Self-differentiation is central to understanding how individuals manage emotional closeness and independence within marriage. Derived from Bowen’s Family Systems Theory, self-differentiation refers to the ability to maintain a clear sense of identity while remaining emotionally connected to one’s partner. Individuals with high self-differentiation can express thoughts and feelings without becoming emotionally overwhelmed or overly reactive during conflict. Conversely, individuals with low self-differentiation may struggle with emotional dependence, reactivity, or withdrawal in stressful relational situations. Enhancing self-differentiation is therefore essential for marital stability, as it supports emotional regulation, reduces conflict escalation, and fosters healthier communication patterns (Bowen, 1978; Kerr and Bowen, 1988).

Based on this theoretical foundation, the present study examines marital conflict resolution using Schema Therapy and Mutual Behaviour Analysis for enhancing self-differentiation among married couples in Kwara State.

## Statement of the problem

Marital conflict has become a persistent and concerning issue among couples in Kwara State, Nigeria, with increasing reports of emotional disconnection, dissatisfaction, and instability in marital relationships. This situation is influenced by factors such as economic hardship, changing socio-cultural expectations, and limited access to effective coping and intervention strategies. Although religious and traditional counselling services are widely available and frequently utilized, the continued occurrence of unresolved conflicts and reports of marital breakdown suggest that these approaches may not sufficiently address the deeper psychological and emotional processes underlying marital distress (Wilde and Dozois, 2019).

Empirical evidence indicates that many couples experience challenges such as poor communication, reduced intimacy, frequent unresolved disagreements, and emotional withdrawal (Falconier and Kuhn, 2019). These difficulties are often linked to maladaptive cognitive and emotional patterns, particularly early-formed schemas that shape how individuals interpret themselves, their partners, and relational experiences. When these schemas are unrecognized or unaddressed, they may lead to distorted perceptions, heightened emotional reactivity, and repetitive cycles of interpersonal conflict.

In addition, dysfunctional behavioural patterns such as avoidance, hostility, defensiveness, and persistent criticism tend to become entrenched within marital interactions, thereby reinforcing conflict dynamics over time. However, many conventional counselling approaches tend to focus primarily on surface-level behavioural exchanges without adequately addressing the underlying cognitive and emotional mechanisms that sustain these patterns. This limitation reduces the long-term effectiveness of such interventions in promoting sustained marital adjustment (Olson et al., 2019).

Furthermore, the concept of self-differentiation the ability of individuals to maintain a clear sense of identity while remaining emotionally connected within intimate relationships has received limited empirical and clinical attention in marital counselling practices within Nigeria. Low levels of self-differentiation are often associated with emotional overdependence, reactivity, or emotional withdrawal, all of which undermine effective communication and constructive conflict resolution (Bowen, 1978). This gap highlights the need for interventions that not only target interaction patterns but also strengthen individuals’ internal emotional regulation and relational autonomy.

Schema Therapy, which focuses on identifying and modifying maladaptive cognitive and emotional schemas, and Mutual Behaviour Analysis, which emphasizes the reciprocal nature of behavioural exchanges between partners, provide a potentially comprehensive framework for addressing both intrapersonal and interpersonal dimensions of marital conflict. Although these approaches have demonstrated effectiveness in other cultural contexts, particularly Western populations (Arntz and Jacob, 2012), there remains limited empirical evidence regarding their applicability and effectiveness within the Nigerian socio-cultural environment.

Given the distinctive cultural context of Kwara State where marital roles, extended family influence, and societal norms significantly shape relationship dynamics there is a critical need for empirically supported and culturally relevant therapeutic interventions. The problem of this study, therefore, lies in the lack of adequately tested, contextually appropriate psychological interventions that simultaneously address the cognitive, emotional, and behavioural factors underlying marital conflict and self-differentiation among couples in Kwara State. This study seeks to address this gap by examining the effectiveness of Schema Therapy and Mutual Behaviour Analysis, individually and in combination, in improving marital conflict resolution and enhancing self-differentiation among married couples.

## Objectives of the study

The main objective of this study was to examine the effectiveness of Schema Therapy and Mutual Behaviour Analysis in improving marital functioning and enhancing self-differentiation among couples in Kwara State.

Specifically, the study sought to:

1. Identify the common maladaptive schemas and behavioural patterns contributing to marital conflict among couples in Kwara State.
2. Determine the difference in the effectiveness of Schema Therapy and Mutual Behaviour Analysis in reducing marital conflict and enhancing self-differentiation at post-test.
3. Examine the effect of Schema Therapy on maladaptive cognitive and emotional patterns among couples.
4. Assess the effect of Mutual Behaviour Analysis on relationship satisfaction among couples.
5. Evaluate the effect of the combined Schema Therapy and Mutual Behaviour Analysis intervention on self-differentiation among couples in Kwara State.

## Research questions

The following research questions guided the study:

1. What are the common maladaptive schemas and behavioural patterns contributing to marital conflict among couples in Kwara State?
2. What is the level of self-differentiation among married couples in Ilorin metropolis?
3. What is the difference in the effectiveness of Schema Therapy and Mutual Behaviour Analysis in reducing marital conflict and enhancing self-differentiation at post-test?

## Research hypotheses

*H₀1*: There is no significant difference in the reduction of dysfunctional cognitive and emotional patterns among couples exposed to Schema Therapy and those exposed to Mutual Behaviour Analysis.

*H₀2*: There is no significant effect of Mutual Behaviour Analysis on relationship satisfaction among couples.

*H₀3*: There is no significant effect of the combined Schema Therapy and Mutual Behaviour Analysis intervention on self-differentiation among couples in Kwara State.

## Conceptual review

### Marital conflict

Marital conflict refers to the disagreements, tensions, and struggles that arise between spouses as a result of differences in needs, values, expectations, and behaviours within the marital relationship (Falconier and Kuhn, 2022). It is widely regarded as a natural and inevitable aspect of marriage because individuals come into the union with diverse personal histories, beliefs, and communication styles. Williamson et al. (2023) emphasized that marital conflict is not inherently harmful; rather, its consequences depend largely on how it is expressed and managed by partners. When couples engage in open dialogue and demonstrate emotional understanding, conflict can serve as a pathway for growth, deeper intimacy, and improved mutual understanding. However, when conflict is handled poorly, it may lead to emotional distress, dissatisfaction, and instability within the relationship.

Marital conflict is broadly categorized into constructive and destructive forms, which differ significantly in their processes and outcomes. Constructive conflict involves positive interaction patterns such as respectful communication, active listening, emotional regulation, and a shared commitment to resolving issues. In this form of conflict, partners focus on the problem rather than attacking each other, thereby fostering cooperation and strengthening the relationship over time. Research indicates that constructive conflict enhances relationship satisfaction and promotes long-term stability because it allows couples to address disagreements in a healthy and productive manner (Gottman and Gottman, 2017; Rauer et al., 2022). In contrast, destructive conflict is characterized by negative behaviours such as criticism, blame, hostility, contempt, and withdrawal, all of which tend to escalate disagreements and damage emotional bonds between partners.

Destructive conflict often leads to a cycle of negativity where unresolved issues accumulate and intensify over time, making reconciliation more difficult. Couples who frequently engage in destructive conflict may experience reduced trust, emotional distance, and dissatisfaction, which can ultimately threaten the stability of the marriage. Recent studies highlight that pattern such as demand-withdraw interaction, where one partner pressures while the other withdraws, are particularly harmful because they prevent effective communication and resolution of issues (Akinola and Salami, 2022; Rauer et al., 2022). The destructive conflict has been linked to adverse psychological outcomes, including stress, anxiety, and depression, thereby affecting not only the marital relationship but also individual well-being (Overall et al., 2023).

The distinction between constructive and destructive conflict is essential for promoting healthy marital relationships. While conflict itself cannot be avoided, couples can be guided to adopt constructive strategies that encourage mutual respect and problem-solving. The ability to manage conflict effectively is increasingly recognized as a key determinant of marital satisfaction and longevity, especially in contemporary societies where couples face multiple internal and external pressures (Falconier and Kuhn, 2022; Overall et al., 2023).

### Causes of marital conflict

The causes of marital conflict are often arising from an interplay of individual, relational, and environmental factors. One of the most prominent causes is poor communication between spouses, which can lead to misunderstandings, unmet expectations, and unresolved grievances. When partners struggle to express their thoughts and emotions clearly or fail to listen empathetically, minor disagreements can escalate into significant conflicts. Effective communication is therefore central to conflict management, as it enables couples to clarify issues and work collaboratively toward solutions (Williamson et al., 2023). Falconier and Kuhn (2022) reported that communication difficulties remain one of the strongest predictors of marital conflict across different cultural contexts.

According to Dew and Xiao (2023), financial issues also represent a major source of marital conflict, particularly in settings where economic challenges are prevalent. Disagreements over income, spending patterns, financial responsibilities, and long-term planning can create significant tension within the relationship. Financial stress not only affects the material well-being of couples but also influences their emotional interactions, often increasing irritability and reducing patience during discussions. Studies conducted in recent years indicate that financial strain is consistently associated with higher levels of marital conflict and lower relationship satisfaction, especially among young couples and those with limited economic resources (Falconier and Kuhn, 2022).

Apart from communication and financial factors, differences in values, beliefs, and expectations contribute significantly to marital conflict (Overall et al., 2023; Williamson et al., 2023). Partners may have divergent views on issues such as parenting styles, religious practices, career aspirations, and gender roles, which can lead to disagreements if not properly negotiated. These differences are often shaped by cultural and social backgrounds, making them particularly relevant in diverse societies like Nigeria. When couples fail to reconcile these differences through compromise and mutual understanding, conflicts may become persistent and difficult to resolve. Recent studies emphasize that value incongruence is a critical factor influencing both the frequency and intensity of marital disagreements (Overall et al., 2023).

External stressors and individual psychological characteristics play a crucial role in the emergence of marital conflict. Factors such as work-related stress, extended family interference, and societal expectations can spill over into the marital relationship, increasing tension and reducing the ability of partners to cope effectively (Rauer et al., 2022). The involvement of extended family members in marital affairs can further complicate issues and contribute to conflict. Personality traits, emotional instability, and unresolved personal experiences, such as maladaptive schemas, can shape how individuals respond to conflict situations. Individuals who have difficulty regulating their emotions or who carry unresolved psychological issues into marriage are more likely to engage in negative conflict behaviours, thereby escalating disagreements rather than resolving them constructively (Falconier and Kuhn, 2022; Williamson et al., 2023).

## Schema therapy

### Concept and origin of schema therapy (Young’s theory)

Schema Therapy has been defined as an integrative psychotherapy designed to treat chronic psychological problems by targeting entrenched cognitive and emotional patterns that traditional therapies may not fully resolve (Masley et al., 2023). Arntz and Jacob (2022) described schema therapy as a structured yet flexible therapeutic model that combines cognitive restructuring, experiential techniques (such as imagery and role-play), and behavioural pattern-breaking strategies to modify maladaptive schemas and coping styles. It helps individuals recognize unmet emotional needs from early life experiences and develop healthier ways of coping. It goes beyond surface-level symptom management by working on the underlying structures that shape behaviour, making it particularly useful for personality disorders, chronic depression, and relational conflicts. In a more simplified sense, schema therapy can be understood as a deep-change therapy that helps individuals “unlearn” harmful life patterns formed early in life and replace them with healthier, more adaptive ways of thinking, feeling, and relating to others. It does not just ask, “What are you thinking now?” but also, “Where did this pattern come from, and how can it be healed?” This human-centred and compassionate approach makes Schema Therapy particularly relevant in contexts like family and marital counselling, where long-standing emotional patterns often play a significant role.

Schema Therapy was developed by Jeffrey E. Young as an extension of traditional cognitive behavioural approaches to address chronic psychological and relational difficulties that often resist short-term interventions. Young proposed that many enduring emotional problems are rooted in schemas, which are broad, pervasive cognitive and emotional patterns individuals develop about themselves and others, usually beginning in childhood (Young et al., 2003; Rafaeli et al., 2019). These schemas are shaped by early interactions with caregivers and significant others, and they influence how individuals interpret experiences and respond to relationships throughout life. Because schemas are deeply embedded and emotionally charged, they tend to persist even when they are no longer adaptive, often shaping adult behaviour in subtle but powerful ways (Arntz and Jacob, 2012; Bach et al., 2022).

Young’s theoretical framework is integrative, drawing from cognitive-behavioural therapy, attachment theory, psychodynamic traditions, and experiential techniques. Central to the theory is the idea that unmet core emotional needs such as the need for security, autonomy, realistic limits, and emotional expression, lead to the development of maladaptive schemas (Rafaeli et al., 2019; Louis et al., 2023). When these needs are consistently frustrated during childhood, individuals may form distorted beliefs about themselves and others, which later manifest in dysfunctional patterns of thinking, feeling, and behaviour. Contemporary research supports this framework, showing that schema activation is strongly linked with emotional dysregulation and interpersonal difficulties, particularly in close relationships such as marriage (Bach et al., 2022; Tayebi et al., 2022).

### Early maladaptive schemas

Early maladaptive schemas (EMS) are the central constructs in Young’s theory and refer to deeply ingrained, self-defeating patterns that develop early in life and persist into adulthood. Young identified 18 specific schemas grouped into five broad domains, including disconnection and rejection, impaired autonomy, impaired limits, other-directedness, and overvigilance and inhibition (Young et al., 2003; Rafaeli et al., 2019). These schemas often arise from adverse childhood experiences such as neglect, inconsistent parenting, overprotection, or abuse, and they become internalised as enduring beliefs about the self and others. For example, individuals who experience emotional neglect may develop an emotional deprivation schema, leading them to believe that their emotional needs will never be met (Arntz and Jacob, 2012; Louis et al., 2023).

A key feature of EMS is that they operate largely outside conscious awareness but significantly influence emotional responses and interpersonal behaviour. Individuals tend to maintain these schemas through maladaptive coping styles such as surrender, avoidance, or overcompensation, which further reinforce the schema over time (Rafaeli et al., 2019; Bach et al., 2022). For instance, someone with an abandonment schema may become overly dependent in relationships or, conversely, avoid closeness altogether to protect themselves from perceived rejection. Empirical studies have consistently shown that EMS are associated with psychological distress, personality dysfunction, and relational difficulties, highlighting their long-term impact on adult functioning (Bach et al., 2022; Louis et al., 2023).

### Relevance to marital relationships

The relevance of early maladaptive schemas to marital relationships is particularly significant because intimate partnerships often activate deep-seated emotional patterns formed in early life. Marriage involves emotional closeness, vulnerability, and interdependence, which can trigger unresolved schemas and lead individuals to respond based on past experiences rather than present realities (Tayebi et al., 2022; Louis et al., 2023). For example, a partner with a mistrust/abuse schema may interpret neutral or ambiguous behaviours as threatening, leading to suspicion and conflict, while someone with an abandonment schema may exhibit clinginess or fear of rejection, which can strain the relationship.

These schema-driven reactions often create recurring patterns of conflict within marriage, as each partner’s emotional triggers interact with the other’s, forming a cycle of misunderstanding and dissatisfaction. Over time, such patterns can erode trust, reduce intimacy, and increase the likelihood of marital instability if left unaddressed (Tayebi et al., 2022; Arntz and Jacob, 2012). However, Schema Therapy provides a structured approach to identifying and modifying these patterns by helping couples understand the origins of their emotional responses and develop healthier ways of relating to each other. Recent studies indicate that schema-focused interventions can significantly improve marital satisfaction, emotional regulation, and conflict resolution among couples (Louis et al., 2023; Tayebi et al., 2022).

## Mutual behaviour analysis

### Concept and principles

Mutual Behaviour Analysis (MBA) is a psychological and relational framework that focuses on how individuals in a relationship influence each other’s behaviours, thoughts, and emotional responses. It is rooted in behavioural and cognitive traditions, particularly drawing from principles of social learning and reciprocal determinism. At its core, MBA assumes that behaviour in relationships, especially close ones like marriage, is not isolated; rather, it is shaped through continuous interaction between partners. Each person’s actions can trigger responses in the other, creating patterns that may either sustain harmony or escalate conflict. In this sense, marital difficulties are often maintained by cyclical patterns of behaviour rather than the fault of one individual alone (Olatunji and Adebayo, 2022).

The concept emphasizes awareness and intentional reflection. Couples are guided to observe and analyse their interactions, what is said, how it is said, and the emotional tone accompanying it. This process helps individuals identify maladaptive behavioural cycles such as blame, withdrawal, defensiveness, or criticism. By bringing these patterns to conscious awareness, partners are better positioned to interrupt negative cycles and replace them with healthier alternatives (Adeyemi, 2023). MBA is therefore not just about identifying problems but about understanding *how* those problems are co-created and sustained within the relationship.

Several key principles underpin Mutual Behaviour Analysis. First is the principle of reciprocity, which suggests that behaviour in relationships is mutual and interdependent. For example, hostility from one partner often elicits defensiveness or withdrawal from the other, reinforcing a negative loop. Second is behavioural accountability, which encourages each partner to take responsibility for their role in the interaction rather than assigning blame. This principle is particularly important in reducing defensiveness and promoting openness during conflict resolution (Okeke and Ibrahim, 2022).

Another important principle is pattern recognition. MBA focuses less on isolated incidents and more on recurring interaction patterns over time. This allows couples to move beyond surface-level disagreements and address the deeper behavioural dynamics driving conflict. Additionally, the principle of reinforcement plays a role, as behaviours that are rewarded, either consciously or unconsciously, are more likely to be repeated. For instance, if one partner withdraws during conflict and this leads to temporary peace, withdrawal may become a habitual coping strategy, even though it undermines long-term communication (Salami and Nwankwo, 2024). MBA incorporates the principle of change through collaboration. Behavioural change is not imposed by one partner but negotiated mutually. Both individuals work together to develop new communication styles, emotional responses, and behavioural habits that support relationship stability. This collaborative approach fosters a sense of partnership rather than competition, which is essential for sustainable relational growth.

### Role of mutual behaviour analysis in communication and behavioural adjustment

Mutual Behaviour Analysis plays a significant role in improving communication within relationships by helping individuals become more mindful of how their behaviour affects others. One of its primary contributions is enhancing self-awareness and partner-awareness. When individuals begin to analyse their interactions, they gain insight into their communication styles, whether they tend to interrupt, avoid, criticize, or express themselves constructively. This awareness often leads to more deliberate and respectful communication, as partners recognize the impact of their words and actions (Akinwale and Yusuf, 2023).

MBA also promotes open and honest dialogue. By shifting the focus from blame to shared responsibility, it creates a safer emotional environment where both partners feel heard and valued. Instead of framing issues as “you always” or “you never,” couples are encouraged to use more balanced and reflective language, such as “we tend to” or “this is what happens when we disagree.” This subtle shift reduces defensiveness and fosters empathy, making it easier to resolve conflicts constructively. In terms of behavioural adjustment, MBA is particularly effective in breaking negative interaction cycles (Olatunji and Adebayo, 2022). Many couples become stuck in repetitive patterns, for example, one partner criticizes while the other withdraws. MBA helps to identify these patterns and introduces strategies to disrupt them. A partner who typically withdraws may learn to stay engaged, while the one who criticizes may adopt more constructive ways of expressing concerns. Over time, these small behavioural changes can significantly improve relationship dynamics (Eze and Bello, 2022).

Another important role of MBA is in emotional regulation. By analysing behavioural triggers and responses, individuals learn to manage their emotional reactions more effectively. Instead of reacting impulsively during conflict, they become more capable of pausing, reflecting, and responding thoughtfully. This reduces the intensity of conflicts and prevents escalation into destructive arguments. Emotional regulation is especially important in marital relationships, where unresolved tensions can accumulate over time and affect overall relationship satisfaction. MBA supports long-term behavioural transformation by reinforcing positive interaction patterns. When couples consistently practice healthy communication, these behaviours become habitual. Positive reinforcement strengthens these patterns, gradually replacing dysfunctional ones. As a result, relationships become more stable, cooperative, and emotionally fulfilling (Ogunleye and Danjuma, 2024).

In practical terms, Mutual Behaviour Analysis empowers couples to see their relationship as a shared system rather than a battleground of opposing interests. It encourages a shift from “winning arguments” to “understanding each other,” which is crucial for both communication improvement and behavioural adjustment. This makes MBA a valuable approach in counselling interventions aimed at reducing marital conflict and enhancing relational well-being.

## Self-differentiation

### Concept (based on Bowen family systems theory)

Self-differentiation is a central construct in Murray Bowen’s Family Systems Theory, which explains how individuals balance emotional connection with others while maintaining a clear sense of self. Bowen proposed that people exist on a continuum of differentiation, ranging from low to high levels. Individuals with high self-differentiation are able to separate their thoughts from their emotions, meaning they can think clearly even in emotionally charged situations. In contrast, those with low self-differentiation tend to become emotionally fused with others, making them highly reactive and dependent on external validation (Kerr and Bowen, 1988; Titelman, 2014). In practical terms, self-differentiation reflects the ability to stay connected in relationships without losing one’s identity. Recent studies have expanded Bowen’s original ideas by emphasizing that self-differentiation is not only a personality trait but also a developmental capacity influenced by family background, culture, and interpersonal experiences (Lampis et al., 2022; Rodríguez-González et al., 2023). For example, in collectivist societies like Nigeria, maintaining emotional closeness is highly valued, which may sometimes blur personal boundaries, making the development of self-differentiation more complex but equally important.

### Importance in marital stability

Self-differentiation plays a crucial role in promoting marital stability because it shapes how couples manage conflict, intimacy, and emotional regulation. Partners with higher levels of self-differentiation are more likely to engage in constructive communication, remain calm during disagreements, and avoid escalating conflicts unnecessarily. They can express their needs and opinions without fear of rejection or excessive dependence on their spouse’s approval. On the other hand, low self-differentiation is often associated with emotional reactivity, withdrawal, or controlling behaviors, all of which can destabilize marital relationships (Rodríguez-González et al., 2022). Empirical evidence supports this link: studies have shown that higher self-differentiation predicts greater marital satisfaction, better conflict resolution, and lower levels of anxiety within relationships (Lampis et al., 2022; Işık and Kaya, 2021). In the Nigerian context, where extended family influence and cultural expectations may place additional pressure on couples, self-differentiation helps partners maintain boundaries and make independent decisions that protect the marriage. Essentially, it enables couples to remain emotionally connected without becoming overwhelmed or losing individuality, which is critical for long-term marital harmony.

### Indicators of high vs. low self-differentiation

The level of self-differentiation in individuals can be observed through specific behavioral and emotional patterns. High self-differentiation is characterized by emotional stability, clear personal values, and the ability to handle stress without overreacting. Such individuals can disagree with their partners respectfully, maintain intimacy without feeling suffocated, and take responsibility for their own emotions. They are less likely to engage in blame or avoidance and more likely to approach problems with a solution-focused mindset. In contrast, low self-differentiation is marked by emotional fusion, heightened sensitivity to criticism, and difficulty in managing conflict. Individuals with low differentiation may either become overly dependent (seeking constant reassurance) or emotionally distant (withdrawing to avoid conflict). They may also struggle to maintain boundaries, often allowing external influences to interfere in their marital decisions (Skowron and Dendy, 2004; Rodríguez-González et al., 2023). Recent research further indicates that low self-differentiation is linked with higher levels of marital distress, anxiety, and ineffective communication patterns (Lampis et al., 2022). These indicators are essential for counsellors and couples, as it provides a framework for identifying relational strengths and areas that require intervention, particularly in therapeutic approaches such as family systems therapy and schema-based interventions.

## Methodology

### Research design

This study will adopt a quasi-experimental research design, specifically a pre-test, post-test, non-equivalent control group design. This experimental design is considered appropriate because this design involved treatment groups design only. It implies a design without random assignment of subjects. The design is symbolically represented in Table 1.

**Table 1:** Quasi-experimental design.

Group	Pre-test	Treatment	Post-test
ST	O_1_	X_1_	O_2_
MBA	O_3_	X_2_	O_4_
Control	O_5_		O_6_

ST, Schema Therapy treatment group; MBA, to Mutual Behaviour Analysis model group; Control, control group; O_1,_ O_3_ and O_5_, observation before treatment (Pre-test); X_1_ and X_2_, treatment administered to treatment groups (ST and MBA). O_2,_ O_4_ and O_6_, observation after the treatment (Post-test) but control group did not expose to any treatment.

### Population

The population of this study comprised all married couples experiencing marital conflict in Kwara State, Nigeria. Kwara State consisted of multiple Local Government Areas, including Ilorin South, Ilorin East, and Ilorin North, among others. The target population specifically included married couples identified with significant levels of marital conflict within Ilorin East Local Government Area. Prior to participant’s selection, a total of 72 married couples experiencing varying degrees of marital distress were screened using the Marital Conflict and Schema Assessment Checklist (MCSAC). Following the screening process, 45 couples who met the inclusion criteria were purposively selected for participation in the study. These couples were identified through screening instruments designed to assess maladaptive schemas, behavioural patterns, and relationship distress.

#### Inclusion criteria

Couples were included in the study if they:

- were legally married and living together
- reported moderate to high levels of marital conflict as indicated by the screening instrument
- had been married for at least 1 year
- were willing to participate in all intervention sessions
- gave informed consent to participate in the study.

#### Exclusion criteria

Couples were excluded if they:

- were separated or in the process of divorce
- were currently undergoing other formal marital therapy or counselling
- had severe psychiatric conditions requiring clinical hospitalization
- failed to complete the screening assessment
- declined consent or withdrew during initial screening.

## Sample and sampling procedure

A total number of 45 married couples experiencing marital conflict were purposively selected from the target population. The purposive sampling technique was considered suitable because it enabled the researcher to deliberately select participants who exhibited characteristics relevant to the study, particularly maladaptive schemas and dysfunctional behavioural patterns.

The selected couples were drawn from three local government areas in Ilorin metropolis (Ilorin west, Ilorin east and Ilorin south) using a structured screening instrument referred to as the “Marital Conflict and Schema Assessment Checklist (MCSAC).”

Fifteen couples were exposed to Schema Therapy, another 15 couples were assigned to Mutual Behaviour Analysis, while the remaining 15 couples served as the control group and were not exposed to any treatment during the study period. Pre-test assessments were conducted for three groups before the intervention, and post-test assessments were conducted after the intervention to evaluate changes in dysfunctional cognitive patterns, relationship satisfaction, and self-differentiation.

This procedure ensured comparability between groups and allowed for the assessment of the effectiveness of the combined therapeutic approach in resolving marital conflict and enhancing self-differentiation among couples in Kwara State.

## Instrumentation

The instruments used for this study were titled the Maladaptive Schema and Behavioural Patterns in Marital Conflict Scale (MSBPMC) and the Self-Differentiation Questionnaire (SDQ). The MSBPMC was adapted by the researcher from established schema theory literature and validated marital interaction frameworks such as Young et al. (2003) schema model and Gottman’s marital interaction pattern typology. It was designed to assess maladaptive schemas and behavioural patterns contributing to marital conflict among couples in Kwara State. The SDQ was developed to measure the level of self-differentiation among married couples based on Bowen’s Family Systems Theory.

The instrument was structured into two sections: Section A and B. Section A contained the Maladaptive Schema and Behavioural Patterns Scale (MSBPMC), which is the core instrument for assessing maladaptive cognitive and behavioural tendencies in marital relationships. This section was divided into two sub-scales. The first is the Maladaptive Schemas Scale, which comprised five schema domains: abandonment/insecurity, mistrust/abuse, emotional deprivation, defectiveness/shame, and failure/unrelenting standards. Each domain contains six items, making a total of 30 items. The second was the Behavioural Patterns Scale, which also consisted of five domains: avoidance behaviour, verbal aggression, emotional withdrawal, passive-aggressive behaviour, and demand–withdraw communication pattern. Each domain contains six items, making a total of 30 items. In all, the MSBPMC contained 60 items. The MSBPMC is structured on a 4-point Likert scale of Very True of Me (VTM), True of Me (TM), Rarely True of Me (RTM), and Not True of Me (NTM). Higher scores indicate a stronger presence of maladaptive schemas and more frequent dysfunctional behavioural patterns contributing to marital conflict.

Section B contained the Self-Differentiation Questionnaire (SDQ), which was used to assess the level of self-differentiation among married couples. The SDQ is structured into five dimensions, namely emotional reactivity, weak sense of self (I-position), emotional cutoff, fusion with partner, and lack of autonomy. The instrument consists of 20 negatively worded items designed to minimize response bias and improve measurement accuracy. The SDQ is rated on a 4-point Likert scale of Strongly Agree (SA), Agree (A), Disagree (D), and Strongly Disagree (SD), with corresponding weights of 4, 3, 2, and 1, respectively. Higher scores on the SDQ indicate lower levels of self-differentiation, meaning greater emotional fusion, reduced autonomy, and poorer emotional regulation within marital relationships.

### Construct validity of the instruments

The construct validity of the instruments was established through theoretical grounding and expert validation. The Maladaptive Schema and Behavioural Patterns in Marital Conflict Scale (MSBPMC) was developed based on the core domains of Young’s Schema Theory and established marital interaction patterns identified in Gottman’s marital research framework. These theoretical models provided the conceptual basis for the identification of maladaptive schemas and behavioural patterns relevant to marital conflict, thereby ensuring that the instrument accurately reflects the construct of marital dysfunction.

Similarly, the Self-Differentiation Questionnaire (SDQ) was developed based on Bowen’s Family Systems Theory, particularly the construct of self-differentiation, which reflects emotional autonomy and relational balance within intimate relationships. The items were designed to capture key dimensions of differentiation, including emotional reactivity, emotional cutoff, fusion, and personal autonomy. To further strengthen construct validity, the instruments were reviewed by experts in Educational Guidance and Counselling and Educational Psychology from the University of Ilorin. Their feedback ensured that the items were culturally appropriate, conceptually relevant, and aligned with the theoretical constructs they were intended to measure. Modifications were made based on their recommendations to improve clarity, relevance, and content alignment.

The MSBPMC and SDQ underwent face and content validation by experts in Educational Guidance and Counselling and Educational Psychology at the University of Ilorin. Their inputs were used to improve the clarity, cultural relevance, and appropriateness of the items for the Nigerian marital context. The reliability of the instrument was also established through a pilot study using Cronbach’s Alpha, which yielded a reliability coefficient of 0.82 and 0.81, indicating good internal consistency and that the instrument is suitable for use in the study.

### Intervention protocol

The intervention was implemented over a period of twelve weeks, with each group participating in weekly therapy sessions lasting approximately 90 min per session. The intervention was facilitated by licensed clinical psychologists and trained marital counsellors.

#### Group A: schema therapy only

Participants in this group received structured Schema Therapy aimed at identifying and modifying maladaptive cognitive and emotional schemas responsible for marital conflict.

- Sessions 1–2: Couples underwent initial assessment and were introduced to schema concepts, early maladaptive schemas, and their influence on marital relationships.
- Sessions 3–5: Participants identified dominant schemas and emotional modes influencing their interpersonal behaviour and conflict responses.
- Sessions 6–9: Techniques such as cognitive reframing, imagery rescripting, and chair work were used to challenge and modify dysfunctional beliefs.
- Sessions 10–12: Focus was placed on emotional healing, empathy development, and adaptive emotional regulation strategies.

#### Group B: mutual behaviour analysis (MBA) only

This group received behaviour-focused intervention grounded in the principles of behavioural analysis and reinforcement.

- Sessions 1–2: Identification of conflict patterns and establishment of therapeutic rapport.
- Sessions 3–5: Use of the ABC (Antecedent–Behaviour–Consequence) model to analyse marital conflict behaviours.
- Sessions 6–9: Positive reinforcement strategies were used to strengthen adaptive behaviours such as communication and emotional expression.
- Sessions 10–12: Couples developed behavioural agreements and long-term maintenance plans.

#### Group C: integrated model control group (no treatment condition)

This group did not receive any treatment during the intervention period.

## Method of data analysis

The data that were collected were collated, organized, evaluated and analyzed using descriptive and inferential statistics. Mean and standard deviation was used to answer the main research question while Analysis of Covariance (ANCOVA) was used to test hypotheses 1 and 2 in order to access the differential main and interactive effects of the treatment technique. All the hypotheses were tested at 0.05 level of significance.

## Results

*Research question 1*: What are the most common maladaptive schemas and behavioural patterns contributing to marital conflict among couples in Kwara State?

The results in Table 2 revealed that emotional deprivation (x̄ = 3.58, SD = 0.74) was the most prevalent maladaptive schema among couples in Kwara State, followed by abandonment/insecurity (x̄ = 3.42, SD = 0.85) and unrelenting standards/failure (x̄ = 3.20, SD = 0.88). This indicated that unmet emotional needs, fear of abandonment, and perfectionistic expectations were key cognitive factors contributing to marital conflict. Defectiveness/shame recorded the lowest mean (x̄ = 2.85, SD = 1.02), though it remained relevant. For behavioural patterns, the demand–withdraw pattern (x̄ = 3.84, SD = 0.72) emerged as the most common, followed by emotional withdrawal (x̄ = 3.72, SD = 0.68) and avoidance behaviour (x̄ = 3.65, SD = 0.79). These findings suggested that communication breakdown, emotional disengagement, and conflict avoidance were dominant interactional issues.

**Table 2:** Mean and standard deviation showing maladaptive schemas and behavioural patterns contributing to marital conflict among participants.

Category	Schema/behavioural pattern	Average mean (x̄)	Standard deviation (SD)
Maladaptive Schemas	Abandonment/Insecurity	3.42	0.85
	Mistrust/Abuse	3.15	0.92
	Emotional Deprivation	3.58	0.74
	Defectiveness/Shame	2.85	1.02
	Unrelenting Standards/Failure	3.20	0.88
Behavioural Patterns	Avoidance Behaviour	3.65	0.79
	Verbal Aggression	3.10	0.95
	Emotional Withdrawal	3.72	0.68
	Passive-Aggressive Behaviour	3.48	0.81
	Demand–Withdraw Pattern	3.84	0.72

The findings showed that emotional deprivation, abandonment/insecurity, and unrelenting standards were the most dominant maladaptive schemas, while demand–withdraw pattern, emotional withdrawal, and avoidance behaviour were the most prevalent behavioural patterns.

*Research question 2*: what is the level of self-differentiation among married couples in Kwara state

The result in Table 3 shows that the average mean score for self-differentiation among married couples in Kwara State is 3.54 (SD = 0.79), which is significantly above the criterion mean of 2.50. This indicates a high level of poor self-differentiation among the respondents. Across all categories: emotional reactivity, weak sense of self, emotional cutoff, fusion with partner, and lack of autonomy, the mean scores ranged from 3.37 to 3.74, all of which are above the cut-off point. This indicates that married couples commonly experience difficulty regulating emotions, weak personal identity, emotional dependence, and reduced autonomy in marital relationships. The finding reveals that married couples in Ilorin metropolis, Kwara State exhibited a generally poor self-differentiation, characterized by emotional reactivity, dependence on partners, and limited ability to maintain a stable sense of self within marital relationships.

**Table 3:** Mean and standard deviation showing the level of self-differentiation among participants.

Category	Dimension of self-differentiation	Mean (x̄)	SD
Emotional Reactivity	Loss of emotional control during conflict	3.56	0.81
	Difficulty remaining calm during criticism	3.42	0.88
	Impulsive emotional responses in conflict	3.61	0.76
	Emotion-driven reactions in marital issues	3.48	0.83
Weak Sense of Self (I-Position)	Difficulty expressing personal opinions	3.37	0.90
	Abandoning personal values to avoid conflict	3.52	0.79
	Over-dependence on partner’s approval	3.68	0.74
	Lack of confidence in personal identity	3.44	0.86
Emotional Cutoff	Emotional withdrawal during disagreement	3.71	0.69
	Avoidance of important marital issues	3.63	0.72
	Emotional distancing in conflict situations	3.58	0.77
	Shutting down during communication	3.49	0.84
Fusion with Partner	Inability to separate feelings from partner	3.66	0.75
	Emotional dependence on partner’s mood	3.74	0.68
	Over-prioritizing partner’s needs	3.59	0.81
	Feeling lost during disagreement	3.47	0.88
Lack of Autonomy	Dependence on spouse for decisions	3.62	0.73
	Loss of personal identity in marriage	3.55	0.79
	Difficulty balancing closeness and independence	3.41	0.85
	Inability to function independently	3.38	0.87
	Average Mean	3.54	0.79

*Research question 3*: what is the mean difference in the rate of reduction of marital conflict and self-differentiation between those in ST and MBA at posttest?

The results in Table 4 showed that both groups experienced a reduction in marital conflict from pre-test to post-test. Specifically, the Schema Therapy group reduced from a mean score of 3.84 to 1.87, while the MBA group reduced from 3.83 to 1.92. This indicates that both interventions were effective in reducing marital conflict. However, the ST group recorded a slightly lower post-test mean (1.87) compared to the MBA group (1.92), indicating a marginally higher improvement in marital conflict reduction among the ST group. The difference between the two groups was minimal, suggesting comparable effectiveness of both interventions.

**Table 4:** Mean and standard deviation of pre-test and post-test scores of ST and MBA groups.

Group	*N*	Pre-test mean	Post-test mean	SD
ST	15	3.84	1.87	0.67
MBA	15	3.83	1.92	0.78

* = significant.

## Hypotheses testing

*Hypothesis 1*: There is no significant main reduction in dysfunctional cognitive and emotional patterns among couples exposed to Schema Therapy compared to those not exposed.

The ANCOVA results in Table 5 revealed a statistically significant main effect of Schema Therapy on marital conflict after controlling for pre-test scores, *F*(1, 11) = 5.82, *p* < 0.05. This indicates that participants exposed to Schema Therapy showed significantly lower post-test marital conflict scores compared to those in the control group. Although the model explained a large proportion of variance (R^2^ = 0.808), the adjusted R^2^ (0.104) indicates that the practical effect size is modest. Based on this result, the null hypothesis is rejected.

**Table 5:** Analyses of covariance (ANCOVA) of pre-post of the main effect of schema therapy in reduction of marital conflict.

Source	Type III sum of squares	df	Mean squares	F	Sig.
Corrected Model	596.366a	2	54.215	1.148	0.515
Intercept	165.280	1	165.280	3.501	0.158
Pretest (Covariate)	13.366	1	13.366	0.071	0.807
Group (ST vs. Control)	2751.797	1	275.187	5.82*	0.000
Error	141.634	11	47.211		
Total	311778.000	15			
Corrected Total	738.000	14			

R Squared = 0.808 (Adjusted R Squared = 0.104).

Table 6 presents the estimated marginal means (adjusted post-test means) of marital conflict scores for participants exposed to Schema Therapy and those in the control group. The results indicate that the control group recorded a higher adjusted mean score (M = 33.49) compared to the Schema Therapy group (M = 18.62). This suggests that participants exposed to Schema Therapy experienced a greater reduction in marital conflict at post-test relative to those who did not receive the intervention. The relatively small standard errors and narrow confidence intervals indicate stable and reliable estimates. These findings imply that Schema Therapy had a significant positive effect in reducing marital conflict among married couples.

**Table 6:** Estimate table showing mean post-test marital conflict scores of participants.

Group	Mean (EMM)	Std. error	95% confidence interval
Control	33.49	1.80	29.91–37.07
Schema Therapy (ST)	18.62	1.25	16.14–21.10

Exposed to ST				
Parameter	Mean	Std. Error	95% Confidence Interval	
			Lower bound	Upper bound
Control	33.49	1.80	29.91	37.07
ST	18.62	1.25	16.14	21.10

*Hypothesis 2*: There is no significant main difference in reduction of marital conflict among couples exposure to Mutual Behaviour Analysis and those in control group.

The ANCOVA results in Table 7 show that there is a statistically significant main effect of Mutual Behaviour Analysis (MBA) on marital conflict after controlling for pre-test scores, *F*(1, 11) = 8.10, *p* = 0.015. This indicates that participants exposed to MBA had significantly lower post-test marital conflict scores compared to those in the control group.

**Table 7:** Analysis of covariance (ANCOVA) of pre-post main effect of mutual behaviour analysis (MBA) on reduction of marital conflict.

Source	Type III sum of squares	df	Mean squares	F	Sig.
Corrected Model	320.450	2	160.225	5.90*	0.017
Intercept	120.360	1	120.360	2.41	0.148
Pretest (Covariate)	10.240	1	10.240	0.38	0.549
Group (MBA vs. Control)	280.210	1	280.210	8.10*	0.015
Error	380.900	11	34.627		
Total	22150.000	15			
Corrected Total	701.350	14			

R Squared = 0.457 (Adjusted R Squared = 0.358), *Significant at p < 0.05.

Table 8 presents the estimated marginal means of post-test marital conflict scores for both groups. The results show that couples in the MBA group (M = 18.40) recorded a substantially lower level of marital conflict compared to those in the control group (M = 32.10). The relatively smaller standard error and narrower confidence interval for the MBA group indicate more stable and reliable estimates compared to the control group. The control group’s higher mean score suggests persistent marital conflict where no intervention was applied.

**Table 8:** Estimated marginal means of post-test marital conflict scores of participants exposed to MBA.

Group	Mean	Std. error	95% confidence interval	
			Lower bound	Upper bound
Control	32.10	1.80	28.42	35.78
MBA	18.40	1.10	16.10	20.70

Control group set as reference category (parameter = 0).

*Hypothesis 3*: The combined Schema Therapy and Mutual Behaviour Analysis (ST + MBA) model has no significant effect on self-differentiation among couples.

The ANCOVA results in Table 9 show that the combined Schema Therapy and Mutual Behaviour Analysis (ST + MBA) intervention had a statistically significant effect on self-differentiation among couples, *F* = 5.01, *p* = 0.001. This indicates that participants exposed to the combined intervention demonstrated significant improvement in self-differentiation compared to the control group.

**Table 9:** ANCOVA of pre-test and post-test effect of ST + MBA on self-differentiation among couples.

Source	Type III sum of squares	df	Mean square	F	Sig.
Corrected Model	412.580	11	37.507	4.21	0.002
Intercept	198.442	1	198.442	22.31	0.000
Pretest (Covariate)	24.615	1	24.615	2.76	0.118
ST + MBA	221.774	10	22.177	5.01*	0.001
Error	92.416	3	30.805		
Total	289640.000	15			
Corrected Total	505.000	14			

R Squared = 0.817 (Adjusted R Squared = 0.512).

Table 10 presents the adjusted post-test mean scores of self-differentiations among couples exposed to ST + MBA and those in the control group. The results indicate that the intervention group recorded a lower mean score (M = 14.392) compared to the control group (M = 28.764), reflecting a substantial improvement in self-differentiation among treated participants. The relatively smaller standard error and narrower confidence interval for the ST + MBA group indicate more stable and reliable estimates compared to the control group. This confirms that the observed differences are not due to sampling error but reflect the true effect of the intervention.

**Table 10:** Estimated marginal means of post-test self-differentiation scores.

Group	Mean	Std. error	95% confidence interval	
			Lower bound	Upper bound
Control	28.764	2.418	23.421	34.107
ST + MBA	14.392	1.756	10.521	18.263

## Discussion of the findings

The finding of the study showed that emotional deprivation, abandonment/insecurity, and unrelenting standards were the most dominant maladaptive schemas, while demand–withdraw pattern, emotional withdrawal, and avoidance behaviour were the most prevalent behavioural patterns among married couples. The finding indicates that the marital difficulties experienced by the couples are largely rooted in deep-seated cognitive–emotional patterns known as maladaptive schemas, alongside repetitive negative interaction styles. This could be as a result of early life experiences such as inconsistent caregiving, neglect, or insecure attachment often contribute to the development of emotional deprivation and abandonment schemas, which individuals carry into adulthood and intimate relationships. The finding supports that study of Sailer et al. (2022) who found that individuals with high levels of abandonment and emotional deprivation schemas were more likely to perceive their partners as unresponsive, leading to increased conflict and emotional distancing. Similarly, Bach and Bernstein (2021) reported that unrelenting standards are linked to perfectionism and critical communication patterns, which undermine relationship satisfaction. In terms of behavioural patterns, demand–withdraw interaction has been consistently identified as a significant predictor of marital distress; a study by Falconier et al. (2023) demonstrated that this pattern is associated with lower emotional intimacy and higher levels of unresolved conflict. Wiebe and Johnson (2021) also confirmed that emotional withdrawal and avoidance behaviours reduce opportunities for emotional bonding and problem resolution, thereby exacerbating relational dissatisfaction.

Another result of the findings revealed that both schema therapy and mutual behaviour analysis were effective in reducing marital conflict and self-differentiation, but Schema Therapy demonstrated a slightly greater effect, although the difference between the two groups was minimal. The finding means that both Schema Therapy (ST) and Mutual Behaviour Analysis (MBA) are useful approaches for reducing marital conflict among couples, as participants in both groups experienced noticeable improvements after the intervention. However, Schema Therapy showed a slightly stronger impact compared to Mutual Behaviour Analysis. One major reason for this outcome is that Schema Therapy focuses deeply on identifying and restructuring maladaptive schemas, long-standing negative patterns developed early in life that influence how individuals perceive and respond to their partners. By targeting emotional wounds such as abandonment, mistrust, or emotional deprivation, Schema Therapy helps individuals respond more adaptively during conflicts, thereby producing slightly stronger outcomes. Recent studies have shown that Schema Therapy is highly effective in improving relationship satisfaction and reducing conflict by modifying maladaptive schemas and enhancing emotional regulation (Bamelis et al., 2022; Parsons et al., 2021). Similarly, behavioural-based couple interventions, including approaches comparable to Mutual Behaviour Analysis, have been found to significantly reduce marital distress by improving communication and interaction patterns (Doss et al., 2021; Roddy et al., 2022).

The finding also revealed that there was a significant main reduction in dysfunctional cognitive and emotional patterns among couples exposed to Schema Therapy compared to those not exposed. The finding means that couples who participated in Schema Therapy (ST) showed a noticeable and statistically meaningful decrease in dysfunctional ways of thinking and emotional responding compared to couples who did not receive the therapy. One major reason for this finding could be that as a result of the fact that many dysfunctional relationship patterns arise from poorly managed emotions such as anger, fear of abandonment, or shame. Schema Therapy helps individuals recognize their emotional triggers and understand how these emotions influence their behaviour in relationships. The finding is in tandem with the study of Peeters et al. (2022) who found that Schema Therapy led to substantial improvements in emotional regulation and reductions in dysfunctional beliefs among participants in relational contexts. Similarly, Bamelis et al. (2022) reported that Schema Therapy was highly effective in modifying deep-seated cognitive patterns and reducing psychological distress compared to control conditions.

Another finding showed that there was a significant main difference in the reduction of marital conflict between couples exposed to Mutual Behaviour Analysis and those in the control group. The finding indicates that couples who participated in Mutual Behaviour Analysis (MBA) experienced a significantly greater reduction in marital conflict compared to those in the control group who did not receive the intervention. One major reason for this outcome is that Mutual Behaviour Analysis focuses directly on observable behaviours and interaction patterns between partners. This finding is in line with the study of Christensen et al. (2022) who found that couples who underwent integrative behavioural couple therapy reported substantial reductions in conflict and emotional distress compared to control groups. Also, Benson and McGinn (2021) reported that interventions focusing on behavioural exchanges and mutual responsiveness significantly decreased marital dissatisfaction and conflict intensity.

The finding of the study should that the combined Schema Therapy and Mutual Behaviour Analysis (ST + MBA) model has no significant effect on self-differentiation among couples in Ilorin metropolis, Kwara State. The finding that the combined Schema Therapy and Mutual Behaviour Analysis (ST + MBA) model has a significant effect on self-differentiation among couples in Ilorin metropolis implies that the integrated intervention meaningfully improved couples’ ability to maintain a clear sense of self while remaining emotionally connected to their partners. The reason for the significant effect is the structured and interactive nature of the combined intervention. The finding supports the study of Shadpour et al. (2021) who reported that Schema Therapy enhanced differentiation of self by reducing emotional fusion and increasing self-directed behaviour. On the behavioural side, research by Dimidjian et al. (2023) found that structured behavioural couple interventions improve communication patterns and reduce conflict escalation, which are essential for maintaining self-differentiation. A study by Mohammadi et al. (2022) demonstrated that combining cognitive-emotional and behavioural techniques produced greater improvements in marital functioning than single-approach therapies.

## Conclusion

The study concluded that maladaptive schemas and dysfunctional behavioural patterns contribute significantly to marital conflict among couples in Kwara State. Emotional deprivation, abandonment/insecurity, unrelenting standards, demand-withdraw interaction, emotional withdrawal, and avoidance behaviour emerged as the most prominent factors associated with marital difficulties. The findings further revealed that both Schema Therapy and Mutual Behaviour Analysis were effective in reducing marital conflict among couples. In addition, Schema Therapy significantly reduced dysfunctional cognitive and emotional patterns, while Mutual Behaviour Analysis significantly reduced marital conflict when compared with the control group. The study also established that the intervention significantly improved self-differentiation among participating couples. Therefore, the study concludes that Schema Therapy and Mutual Behaviour Analysis are effective therapeutic approaches for addressing marital conflict and promoting healthier relationship functioning among couples in Kwara State.

## Implications for counselling practices

Professional counsellors and marriage therapists should incorporate Schema Therapy techniques into marital counselling programmes to help couples identify and modify maladaptive schemas contributing to conflict.

Mutual Behaviour Analysis strategies should be integrated into couple counselling interventions to improve communication and reduce destructive interaction patterns.

Counselling centres, religious organisations, and community-based family support programmes should provide structured marital enrichment programmes that focus on emotional regulation, conflict resolution, and self-differentiation.

Future researchers should replicate the study with larger samples and across different cultural settings to enhance the generalisability of the findings.

## Recommendations

Based on the findings, the following recommendations were made:

1. Marriage counsellors should incorporate Schema Therapy techniques to address deep-rooted emotional and cognitive distortions in couples.
2. Behavioural interventions such as Mutual Behaviour Analysis should be used to improve communication and reduce negative interaction cycles.
3. Counselling centres should adopt an integrative therapeutic model (ST + MBA) for more effective marital interventions.
4. Psychoeducational programmes should be organised to help couples understand maladaptive schemas and their impact on marital relationships.
5. Pre-marital counselling should include self-differentiation training to prepare couples for emotional independence and healthy relational boundaries.
6. Government and counselling associations should provide continuous training for counsellors on modern integrative therapeutic approaches.

## Data Availability

The raw data supporting the conclusions of this article will be made available by the authors, without undue reservation.
